# Haglund’s Syndrome: A Commonly Seen Mysterious Condition

**DOI:** 10.7759/cureus.820

**Published:** 2016-10-07

**Authors:** Raju Vaishya, Amit Kumar Agarwal, Ahmad Tariq Azizi, Vipul Vijay

**Affiliations:** 1 Orthopaedics, Indraprastha Apollo Hospitals; 2 Orthopaedics, Herat Regional Hospital, Herat, Afghanistan

**Keywords:** haglund's syndrome, retrocalaneal exostosis

## Abstract

Haglund’s deformity was first described by Patrick Haglund in 1927. It is also known as retrocalcaneal exostosis, Mulholland deformity, and ‘pump bump.' It is a very common clinical condition, but still poorly understood. Haglund’s deformity is an abnormality of the bone and soft tissues in the foot. An enlargement of the bony section of the heel (where the Achilles tendon is inserted) triggers this condition. The soft tissue near the back of the heel can become irritated when the large, bony lump rubs against rigid shoes. The aetiology is not well known, but some probable causes like a tight Achilles tendon, a high arch of the foot, and heredity have been suggested as causes. Middle age is the most common age of affection, females are more affected than males, and the occurence is often bilateral. A clinical feature of this condition is pain in the back of the heel, which is more after rest. Clinical evaluation and lateral radiographs of the ankle are mostly enough to make a diagnosis of Haglund’s syndrome. Haglund’s syndrome is often treated conservatively by altering the heel height in shoe wear, orthosis, physiotherapy, and anti-inflammatory drugs. Surgical excision of the bony exostoses of the calcaneum is only required in resistant cases.

## Introduction and background

Haglund’s deformity was first described by Patrick Haglund in 1927 [1]. It is also known as retrocalcaneal exostosis, Mulholland deformity, and ‘pump bump.' Although a very common clinical condition, it is still poorly understood. It has no definitive etiology, but various probable causes like a tight Achilles tendon, a high arch of the foot, and heredity have been proposed. It usually affects middle-aged people, females more than males, and it is often bilateral. It is characterized by pain in the back of the heel, which is more after rest. Clinical evaluation and lateral radiographs of the ankle are mostly enough to make a diagnosis of Haglund’s syndrome. The pain could be due to associated Achilles tendonitis and retrocalcaneal bursitis [2]. This condition can mimic other causes of hind foot pain like isolated retrocalcaneal bursitis, plantar fasciitis, and seronegative spondyloarthropathies [3].

Haglund’s syndrome is often treated conservatively by altering the heel height in shoe wear, orthosis, physiotherapy, anti-inflammatory drugs, and local steroid injection. Surgical excision of the bony exostoses of the calcaneum is only required in resistant cases.

## Review

Haglund’s deformity is an abnormality of the posterosuperior part of the calcaneus, where there is a bony enlargement at the attachment of the Achilles tendon. The adjoining soft tissues can get irritated when this bony lump rubs against rigid shoes. It often leads to retrocalcaneal bursitis, calcaneal tendon bursitis, and thickening and inflammation of the calcaneal tendon. This combination of pathology is known as Haglund’s syndrome. Inflammation of the different parts of soft tissue in the area can lead to an isolated condition; however, the treatment options are different in these conditions, and so they should be differentiated.

### Cause

It is mostly an idiopathic condition, but several contributory factors like over-practice in runners, tight or poorly fitting shoes, or altered biomechanics of foot joints because of the dealigned subtalar joint may play a role [4].

### History

Pain at the posterior heel is the presenting feature. It may be associated with limping and swelling. The pain is prominent while the patient begins to walk after rest. This condition may be unilateral or bilateral. History of any rheumatologic conditions like gout, rheumatoid arthritis, or seronegative spondyloarthropathies should be considered. [5].

### Physical examination

**Figure 1:**
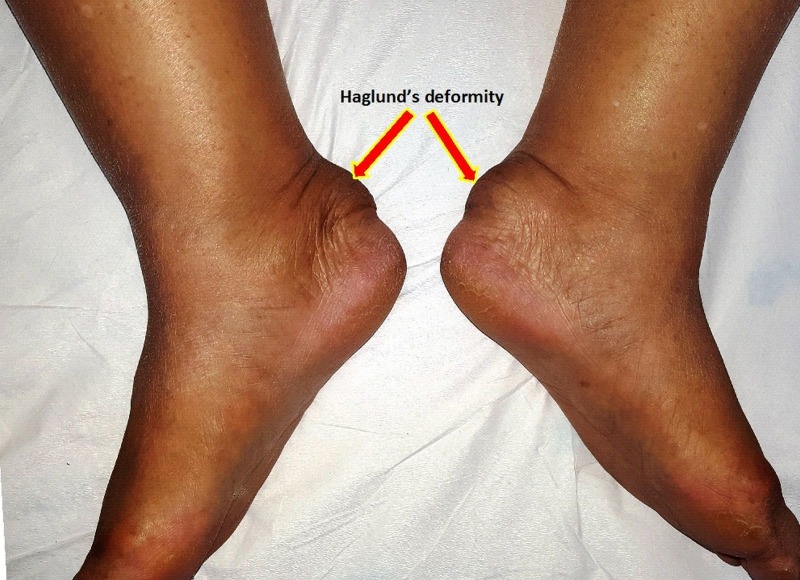
Clinical photograph of both ankles showing prominent swelling over the back of both heels

A bump is seen on the posterior heel (Figure 1).

Signs of inflammation like swelling, warmth, redness, and tenderness may be present over the posterior heel. If a careful physical examination is done, it may be possible to differentiate whether the inflammation is anterior to the Achilles tendon or posterior.

### Radiographic evaluation

**Figure 2:**
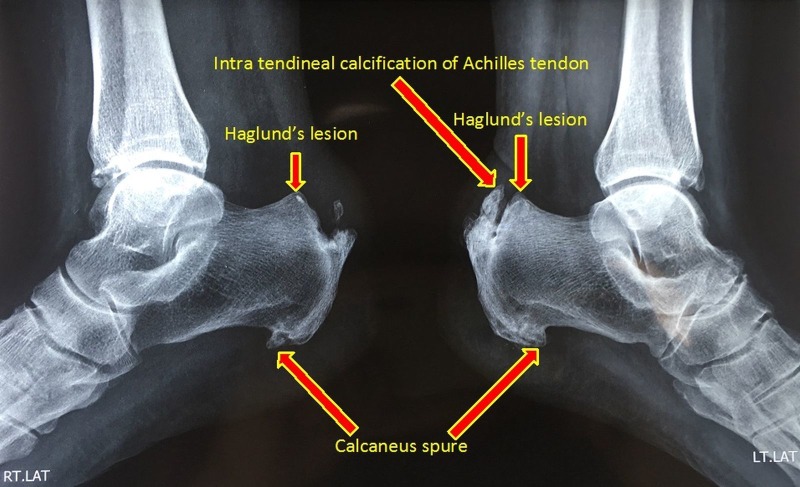
Lateral radiograph of ankles showing bony enlargement at the posterosuperior aspect of both calcaneum

In a lateral radiograph, a bony prominence (Haglund's lesion) at the posterosuperior part of the calcaneal tuberosity, calcaneal bursal swelling, and increased density in pre-Achilles bursae (Figure 2) are apparent in these patients [6]. These findings may be associated with a calcaneal spur and heterotopic bone formation at the insertion of the Achilles tendon and within the Achilles tendon. The diagnosis of Haglund's syndrome is often based on history and clinical findings; radiographic changes may add a further clue to its diagnosis. There are no clear cut radiological criteria for diagnosing Haglund's lesion, especially in the early stages. However, some angles on plain radiographs have been described [7-8].

**Figure 3:**
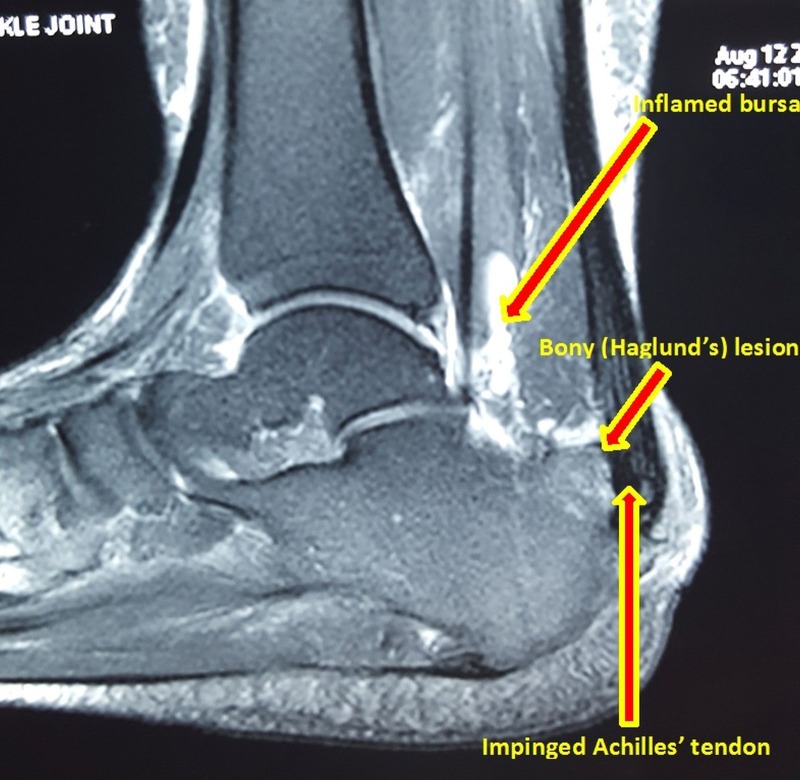
MRI image of ankle and foot showing posterosuperior bony spurring of calcaneus, retrocalcaneal bursitis, and impingement of Achilles tendon

A magnetic resonance imaging (MRI) scan is done in questionable cases. It shows posterosuperior calcaneal spurring with impingement on the Achilles tendon. There may be associated synovial thickening and collection in the retrocalcaneal bursa with thickening and high signal in the insertional fibers of Achilles tendon and edema in the adjoining retro-Achilles fat pad (Figure 3). All these findings are consistent with Achilles tendinosis with retrocalcaneal and retro-Achilles bursitis [9].

### Differential diagnosis 

Detection of localized tenderness around the heel may help in differentiating this syndrome from mimicking conditions like isolated calcaneal bursitis, insertional Achilles tendinosis, plantar fasciitis, and avulsion of the calcaneal tendon (Table 1).

**Table 1:** Distinguishing features of the conditions mimicking Haglund’s syndrome

Conditions	Signs and symptoms
Calcaneal bursitis	Tenderness is mostly palpable on medial or lateral sides and in front of Achilles tendon.
Achilles tendinosis	Tenderness is present distally at the insertion of the Achilles tendon on the Calcaneus.
Planter fasciitis	Pain and tenderness is most commonly over the sole of the feet.
Avulsion of calcaneal tendon	A defect of gap is palpable in the tendon and Thompson test is positive.

### Treatment

Conservative measures include a reassessment of the shoe of the patient and heel pads or heel lifts in the cases of high arched feet [10]. Casting may be necessary for pain reduction and an ice bag may be necessary to deal with swelling. Anti-inflammatory drugs (oral or topical), stretching exercises, and physiotherapy may relieve tension from the calcaneal tendon. Local perilesional steroid injections are also used in refractory cases [11].

If conservative treatment is not effective then surgical treatment options like retrocalcaneal decompression and calcaneal ostectomy or osteotomy are used [12]. Inadequate bone resection can lead to the recurrence of symptoms [13].

**Figure 4:**
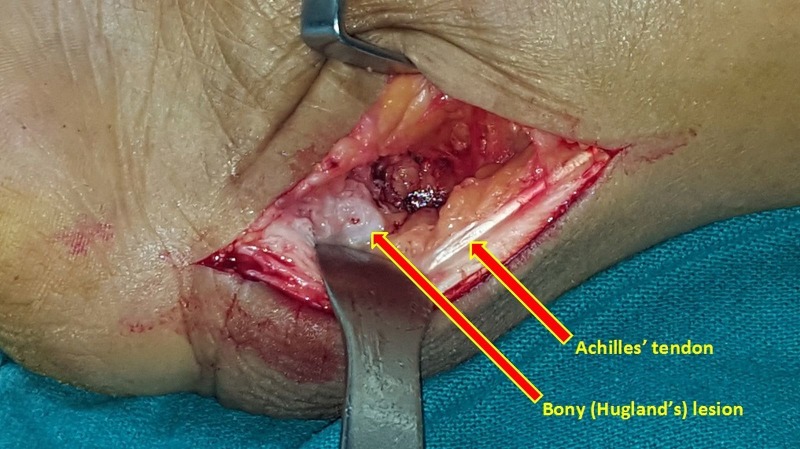
Intraoperative image of the bony lesion in Haglund’s syndrome

The surgical technique is as follows. After administration of general or regional anesthesia, a longitudinal lateral incision 1 cm lateral to the Achilles tendon is made, extending distally from 3–4 cm proximal to the superior tuberosity of the calcaneus to 2–3 cm distal to the superior tuberosity of the calcaneus. The ankle joint is plantar flexed, and by sharp and blunt dissection, the Achilles tendon is identified. A right-angled retractor is placed between the Achilles tendon and the posterior and superior borders of the calcaneal tuberosity. The retrocalcaneal bursa is exposed as well as the superior border of the calcaneal tuberosity without raising any of the Achilles tendon off the calcaneus (Figure 4).

**Figure 5:**
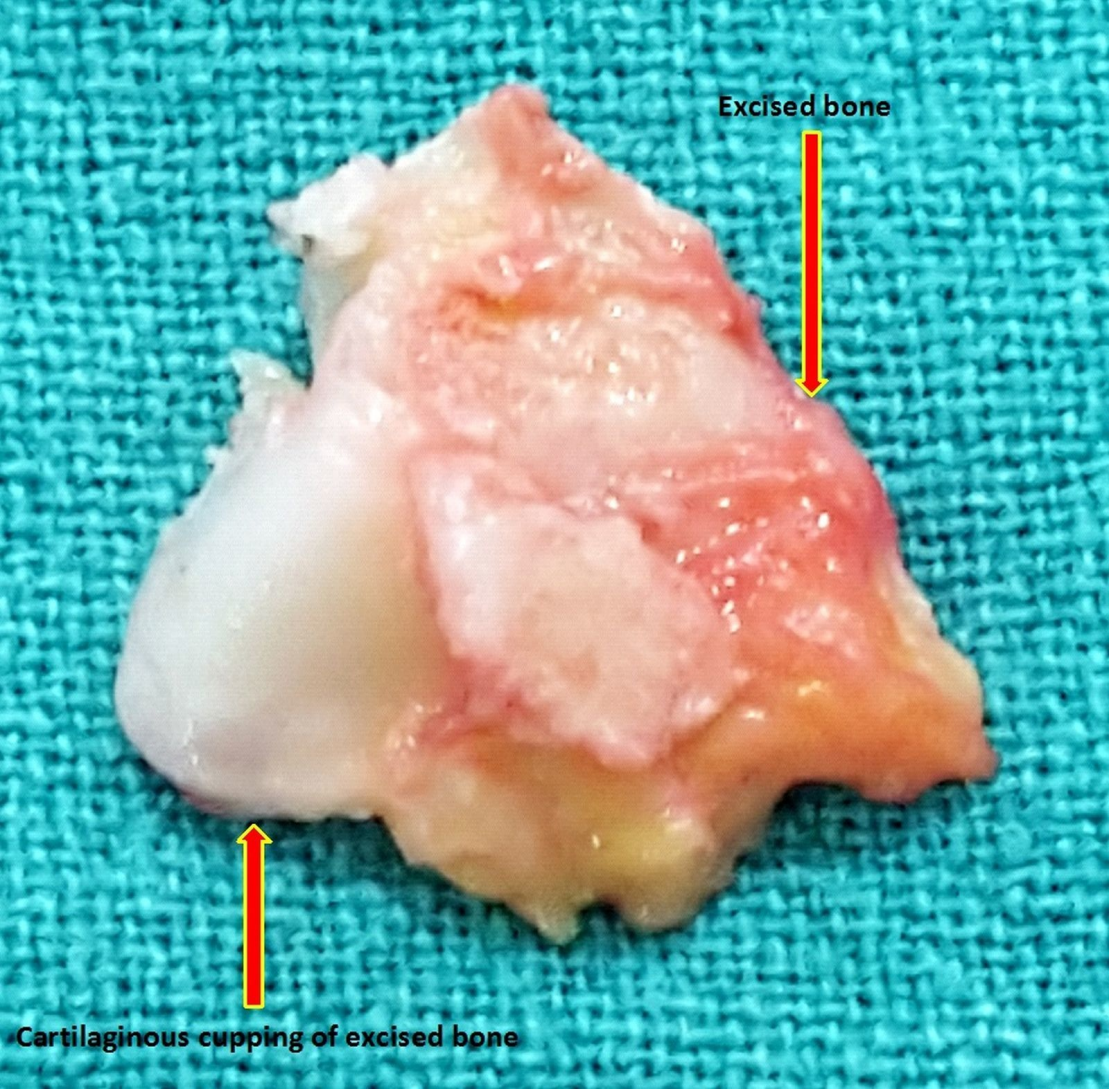
Excised bony (Haglund’s) lesion

However, the Achilles tendon has such an extensive insertion into the posterior and plantar aspect of the calcaneal tuberosity that raising a 1–2 cm-long portion of the tendon may be necessary to resect the bone adequately. The retrocalcaneal bursa is removed first then the superior aspect of the tuberosity is removed with an osteotome (Figure 5). The placement of several drill holes along the proposed osteotomy site makes this resection easier. If intratendineal calcification is present, it should be removed. A well-padded, short leg, non-weight-bearing cast is applied, with the ankle in approximately 20 degrees of plantar flexion.

The cast and sutures are removed at two weeks, but the non-weight-bearing cast remains on for three weeks. Then a removable weight-bearing cast boot is applied, and active plantar flexion and dorsiflexion exercises are begun. It is important in the preoperative counseling to explain to a young woman with a pump bump that it might be three to six months before she can wear a stylish shoe and that there is no guarantee that she will ever be able to do so comfortably [14].

The reported surgical complications include Achilles tendon avulsion, persistent posterior heel pain, wound breakdown, nerve injuries (medial calcaneal sensory nerve and sural nerve), ankle stiffness, and incisional neuroma [15].

## Conclusions

Haglund’s syndrome is a common cause of hind foot pain in adults, but it is still a poorly understood clinical condition. Conservative management is often effective in most cases, and surgery is required only in resistant cases.
